# Correction: Thrombotic and bleeding events, mortality, and anticoagulant use among 546,656 hospitalized patients with COVID‑19 in the United States: a retrospective cohort study

**DOI:** 10.1007/s11239-022-02708-3

**Published:** 2022-09-19

**Authors:** Steve Deitelzweig, Xuemei Luo, Jennifer L. Nguyen, Deepa Malhotra, Birol Emir, Cristina Russ, Xiaoyan Li, Theodore C. Lee, Mauricio Ferri, Danny Wiederkehr, Maya Reimbaeva, Geoffrey D. Barnes, Gregory Piazza

**Affiliations:** 1 Ochsner Clinic Foundation, Department of Hospital Medicine, Ochsner Medical Center, The University of Queensland School of Medicine, Ochsner Clinical School, 1514 Jefferson Hwy, New Orleans, LA 70121 USA; 2 Pfizer Inc., Groton, CT USA; 3 Pfizer Inc., New York, NY USA; 4 Bristol Myers Squibb Company, Lawrenceville, NJ USA; 5 Frankel Cardiovascular Center, University of Michigan, Ann Arbor, MI USA; 6 Brigham and Women’s Hospital, Boston, MA USA

## Correction to: Journal of Thrombosis and Thrombolysis (2022) 53:766–776 10.1007/s11239-022-02644-2

The article “Thrombotic and bleeding events, mortality, and anticoagulant use among 546,656 hospitalized patients with COVID‑19 in the United States: a retrospective cohort study”, written by Steve Deitelzweig, Xuemei Luo, Jennifer L. Nguyen, Deepa Malhotra, Birol Emir, Cristina Russ, Xiaoyan Li, Theodore C. Lee, Mauricio Ferri, Danny Wiederkehr, Maya Reimbaeva, Geoffrey D. Barnes and Gregory Piazza was originally published electronically on the publisher’s internet portal on 30 April 2022 without open access. With the author(s)’ decision to opt for Open Choice the copyright of the article changed on 30 August 2022 to © The Author(s) 2022 and the article is forthwith distributed under a Creative Commons Attribution 4.0 International License, which permits use, sharing, adaptation, distribution and reproduction in any medium or format, as long as you give appropriate credit to the original author(s) and the source, provide a link to the Creative Commons licence, and indicate if changes were made. The images or other third party material in this article are included in the article’s Creative Commons licence, unless indicated otherwise in a credit line to the material. If material is not included in the article’s Creative Commons licence and your intended use is not permitted by statutory regulation or exceeds the permitted use, you will need to obtain permission directly from the copyright holder. To view a copy of this licence, visit http://creativecommons.org/licenses/by/4.0.

